# Characterizing cognitive workload during simulated surface extravehicular activity with integrated virtual reality

**DOI:** 10.3389/fpsyg.2026.1713354

**Published:** 2026-01-26

**Authors:** Steven R. Anderson, Crystal Kirkley, Alex J. Baughman, Caleb Cram, Eryn Andrews, Kyoung Jae Kim, Rebecca DiDomenica, Morgan Stosic, Bradley Hoffmann, Patrick Estep, Suzanne T. Bell, Karina Marshall-Goebel, Daniel M. Buckland

**Affiliations:** 1 Behavioral Health and Performance Laboratory, Biomedical Research and Environmental Sciences Division, Human Health and Performance Directorate, NASA Johnson Space Center, KBR, Inc., Houston, TX, United States; 2 Fatigue Countermeasures Laboratory, Human Systems Integration Division, NASA Ames Research Center, National Aeronautics and Space Administration (NASA), Mountain View, CA, United States; 3 EVA and Environmental Physiology Laboratory, Biomedical Research and Environmental Sciences Division, Human Health and Performance Directorate, NASA Johnson Space Center, GeoControl Systems, Houston, TX, United States; 4 EVA and Environmental Physiology Laboratory, Biomedical Research and Environmental Sciences Division, Human Health and Performance Directorate, NASA Johnson Space Center, Aegis Aerospace, Houston, TX, United States; 5 EVA and Environmental Physiology Laboratory, Biomedical Research and Environmental Sciences Division, Human Health and Performance Directorate, NASA Johnson Space Center, KBR, Inc., Houston, TX, United States; 6 Behavioral Health and Performance Laboratory, Biomedical Research and Environmental Sciences Division, Human Health and Performance Directorate, NASA Johnson Space Center, JES Tech, Houston, TX, United States; 7 Behavioral Health and Performance Laboratory, Biomedical Research and Environmental Sciences Division, Human Health and Performance Directorate, NASA Johnson Space Center, National Aeronautics and Space Administration (NASA), Houston, TX, United States; 8 EVA and Environmental Physiology Laboratory, Biomedical Research and Environmental Sciences Division, Human Health and Performance Directorate, NASA Johnson Space Center, National Aeronautics and Space Administration (NASA), Houston, TX, United States; 9 Department of Emergency Medicine and Mechanical Engineering, University of Wisconsin-Madison, Madison, WI, United States

**Keywords:** cognitive workload, human spaceflight, surface extravehicular activity, task performance, virtual reality

## Abstract

**Introduction:**

High cognitive workload presents an important risk to crew safety during surface exploration extravehicular activity (EVA). However, it remains difficult to draw conclusions about the precise EVA task characteristics that are associated with increases in cognitive workload, or their relationship to performance.

**Methods:**

In the present study, we examined how experimentally manipulating cognitive workload during a surface EVA task was related to subjective cognitive workload assessments, physiological responding, and EVA performance outcomes. Participants (N = 14) completed surface EVA simulations using a virtual reality and integrated treadmill setup in an extended reality exploration surface analog.

**Results:**

Experimentally manipulating the difficulty of geological sample identification was associated with increased subjective cognitive workload, decreased cognitive performance, altered physiological responding, and decreased performance on key EVA tasks. Results demonstrate evidence for a relationship between the cognitive demands of a surface EVA task and the subjective and physiological correlates of cognitive workload and cognitive performance, as well as decrements in EVA task performance due to increased cognitive demand.

**Discussion:**

Cognitive workload during surface exploration EVA may be an important consideration for EVA scheduling and monitoring during critical mission tasks on the Moon and Mars.

## 1 Introduction

Surface extravehicular activity (EVA) places humans outside the safety of a vehicle and closer to exploration targets than any other endeavor in space. Despite providing crucial firsthand experience of another planetary body, there are also risks to astronaut health and safety associated with surface EVA. The NASA Human System Risk Board has defined the “Risk of Mission Impacting Injury and Compromised Performance and Long-Term Health Effects due to EVA Operations”^1^ as having a likelihood and consequence score of 5 × 4 (i.e., >50% likelihood and consequence of severe reduction of crew performance that results in loss of multiple mission objectives), a red-category risk requiring mitigation during lunar orbital/surface and Mars surface EVA. Compared to microgravity EVA conducted from the ISS and Apollo-era partial gravity EVA conducted on the lunar surface, future partial gravity surface EVA on the Moon and Mars is likely to have increased operational tempo, increased cognitive and physical workload, increased probability of injuries and performance decrements, as well as more crew autonomy and flexibility in executed timelines (Dunn et al., 2022). Specific risks of surface EVA include the increased risk of lower extremity abrasions, overuse injuries resulting from the planned cadence of Artemis EVA, and the long-term effects of partial gravity, which are still not well understood (Richter et al., 2017).

In addition to the physical demands, a small but growing body of evidence has documented the cognitive demands associated with surface EVA (Anderson et al., 2026; Logie, 2011) in ground-based EVA simulation environments. These include NASA’s Neutral Buoyancy Laboratory (NBL), Active Response Gravity Offload System (ARGOS), NASA Extreme Environment Mission Operation (NEEMO) undersea missions, Joint EVA & Human Surface Mobility Test Team (JETT) field environments, and virtual reality (VR). NASA’s JETT field tests have documented instances where physical workloads exceeded the 90% age-predicted heart rate maximum value (primarily due to pushing the tool cart needed for field geology), and cognitive workload values indicating limited spare cognitive capacity as measured by the Bedford Workload Scale (Coan and Miller, 2023). Similarly, during EVA simulation conducted in the Mars Desert Research Station, average subjective cognitive workload as measured by the NASA Task Load Index (TLX) was significantly higher during EVA tasks compared to hill runs (Rai et al., 2012). In an assessment of metabolic rate (MR) in a suited, partial-gravity (1/6 G) EVA simulation conducted in ARGOS, participants had high MR (defined as ≥1,000 BTU/h) in 47% of end-to-end geology sampling EVA (Schlotman et al., 2023). Cognitive workload assessed during simulated EVA conducted as part of an undersea NEEMO mission indicated changes in subjective workload during different simulated EVA tasks and decrements in team functioning associated with high cognitive workload (Ari et al., 2020). Insights derived from these simulation environments can ultimately be used to inform concept of operations (ConOps) and planning for future surface EVA on the Moon and Mars.

With the goal of characterizing the specific cognitive demands of surface EVA tasks, NASA’s Behavioral Health & Performance Laboratory recently conducted a cognitive task analysis of surface EVA with astronauts and EVA subject matter experts (Anderson et al., 2026). Experts indicated that they anticipated high cognitive demand during several surface EVA tasks, in particular geological sample identification and traverse. Additionally, a cognitive domain that emerged from this cognitive task analysis as crucial for optimal surface EVA performance was working memory (Logie, 2011), which enables astronauts to keep track of ongoing mental processes related to the multiple science, system, and safety tasks present during surface EVA.

Although EVA simulation and cognitive science studies to date provide valuable insight on the tasks that may influence cognitive workload during surface EVA, it remains difficult to draw conclusions about the precise EVA task characteristics that are associated with increases in cognitive workload, as well as how increases in cognitive workload predict EVA performance. As a result, there is a need to experimentally manipulate cognitive workload—while holding other relevant factors such as physical workload constant—in EVA simulation studies so that we can understand the unique risks presented by cognitive workload during surface EVA. The purpose of the present study was to examine the extent to which experimentally manipulating cognitive workload in a surface EVA task was related to subjective cognitive workload assessments, physiological correlates, and EVA performance outcomes.

To accomplish this, we manipulated cognitive workload during simulated geological sample identification in an extended reality exploration EVA analog, NASA’s Assessments of Physiology and Cognition in Hybrid-reality Environments (APACHE) facility. We designed our experimental manipulation of the primary task of geological sample identification to increase demands on working memory specifically, following the findings of our previously conducted surface EVA cognitive task analysis (Anderson et al., 2026). Drawing upon the extensive literature on working memory demands during dual-task paradigms, in which consistent decrements in performance have been found with increasing working memory load and cognitive task complexity (Baddeley, 2017; Della Sala et al., 1995; Gathmann et al., 2015; Lee and Kang, 2002), and with the goal of increasing EVA simulation fidelity, we also included a secondary task of self-spacesuit temperature monitoring and a tertiary task of an unexpected geological discovery. We included measures of physiological responding (heart rate, heart rate variability) and fatigue given that prior experimental studies indicate that task-related stress and cognitive workload typically increases heart rate (Schubert et al., 2009) and decreases HRV (Delliaux et al., 2019; Hughes et al., 2019; Schubert et al., 2009), likely due to increased sympathetic nervous system activation and parasympathetic withdrawal (Castaldo et al., 2015; Anderson et al., 2026; Della Sala et al., 1995; Gathmann et al., 2015; Lee and Kang, 2002). Our manipulation check and hypotheses are below.

*Hypothesis 1:* Compared to a low cognitive workload task, a high cognitive workload task will be associated with decrements in cognitive performance measures (Cognition Test Battery, VR Digit-Symbol Substitution Test, Auditory Reverse Digit Span Test).

*Hypothesis 2:* Compared to a low cognitive workload task, a high cognitive workload task will be associated with increased heart rate (HR), decreased heart rate variability (HRV), and increased fatigue.

*Hypothesis 3:* Compared to a low cognitive workload task, a high cognitive workload task will be associated with decrements in performance on the primary EVA task of geological sample identification (quality, efficiency, completion time).

*Hypothesis 4:* Compared to a low cognitive workload task, a high cognitive workload task will be associated with decrements in performance on a secondary EVA task of self-spacesuit temperature monitoring (quality, situational awareness).

*Hypothesis 5:* Compared to a low cognitive workload task, a high cognitive workload task will be associated with decrements in performance on a tertiary EVA task of an unexpected geological discovery (likelihood of noticing).

*Manipulation check:* Compared to a low cognitive workload task, a high cognitive workload task will be associated with increases in subjective cognitive workload measures (NASA-TLX, Bedford Workload Scale).

## 2 Materials and methods

Testing was completed from December 2024 to April 2025 in the APACHE facility at NASA Johnson Space Center in Houston, TX, USA. Each participant completed study sessions in the following order: (1) Study Briefing (~1 h) conducted online, (2) Familiarization Session (~4 h) conducted in person in the APACHE facility, and (3) Test Session conducted in person in the APACHE facility comprising a morning and afternoon virtual reality (VR) EVA (~3 h each; ~6 h total) with a 1-h break between morning and afternoon sessions. Test Sessions involved completing one low and one high cognitive workload VR EVA segment in a randomized and counterbalanced fashion. The Study Briefing, Study Familiarization, and Test Sessions were completed on separate days.

### 2.1 Participants

#### 2.1.1 Recruitment

Participants were recruited through NASA Test Subject Screening. The inclusion criterion was that a participant must be a healthy adult with no orthopedic limitations for standing/walking for long periods of time. The exclusion criterion was susceptibility to motion sickness as measured by the short form of the Motion Sickness Susceptibility Questionnaire (MSSQ – Short). The MSSQ – Short includes items asking about the frequency of motion sickness in different scenarios experienced in childhood and in the last 10 years (Golding, 2006).

#### 2.1.2 Power analysis

A simulation-based power analysis for linear mixed models was conducted using the *SIMR* (Green and MacLeod, 2016) and *mixedpower* (Kumle et al., 2021) packages in R (Green and MacLeod, 2016; Kumle et al., 2021). The power analysis indicated sufficient power (0.80) at a sample size of at least *N* = 12 participants based on medium-to-large (*d* = 0.5–0.8) effect sizes reported in meta-analyses of the effect of cognitive workload manipulations on self-reported (Hertzum, 2021; Shahini and Zahabi, 2022) and physiological (Hughes et al., 2019) measures of cognitive workload. To account for possible dropout due to cybersickness or other technical issues, data from a total of 16 participants was collected. Data from two participants were excluded from further analyses for the following reasons: one participant completed a familiarization session but did not complete a test session due to cybersickness; one participant completed a familiarization session and morning test session but did not complete an afternoon test session due to cybersickness. After excluding participants who did not successfully complete a test session, a final sample size of *N* = 14 was achieved. Demographic characteristics for participants included in the final sample are in Table 1.

**Table 1:** Subject demographic characteristics.

Demographics	Total (*N* = 14)
Age (Years)	
Mean (SD)	42.4 (11.3)
Median [Min, Max]	39.0 [26.0, 61.0]
Gender	
Female	5 (35.7%)
Male	9 (64.3%)
Education	
4-Year college degree	2 (14.3%)
Master’s degree	9 (64.3%)
Doctorate degree	1 (7.1%)
Missing	2 (14.3%)
Exercise	
Once or more per day	1 (7.1%)
4–5 times per week	5 (35.7%)
2–3 times per week	5 (35.7%)
2–3 times per month	1 (7.1%)
Missing	2 (14.3%)
Simulated EVA experience (% Yes)	
Virtual Reality (VR)	6 (42.9%)
ARGOS	3 (21.4%)
NBL	5 (35.7%)
Field test	2 (14.3%)
Hypobaric chamber	4 (28.6%)
Other Analog	3 (21.4%)
No Experience	3 (21.4%)

Participants could indicate more than one category of EVA Experience.

### 2.2 APACHE VR environment integrated with treadmill

**Figure 1:**
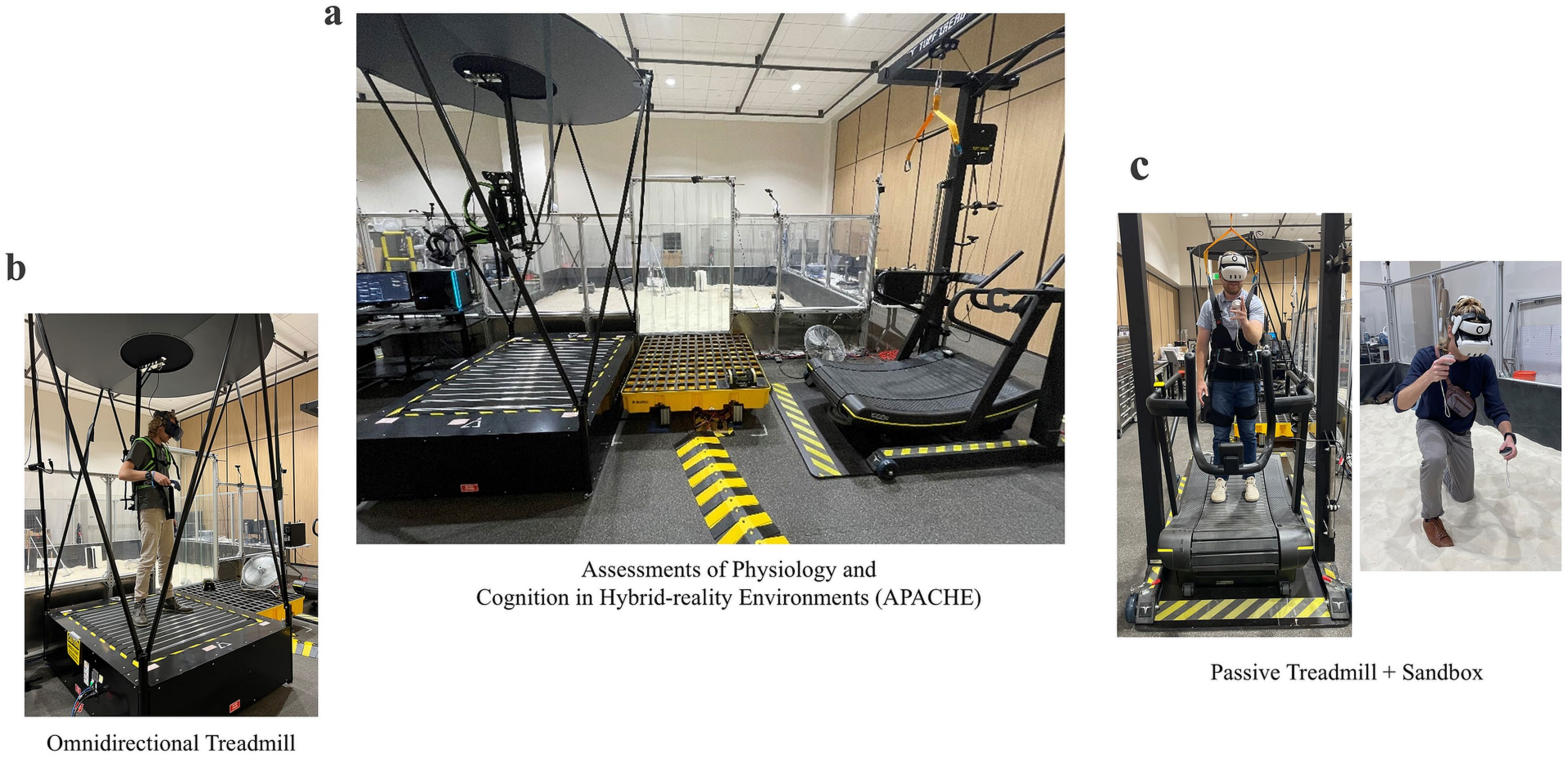
Assessments of Physiology and Cognition in Hybrid-reality Environments (APACHE). **(a)** View of APACHE space; **(b)** Omnidirectional treadmill; **(c)** Passive treadmill and sandbox configuration. The individuals pictured are co-authors of the present study (AB and CC) and were not research participants.

All data collections were performed at NASA Johnson Space Center (JSC) in the hybrid-reality APACHE test environment (Figure 1a). In the first half of data collection (*N* = 8), participants walked on the Infinadeck omnidirectional treadmill (Infinadeck, Rocklin, CA, USA), a ~1.22 × 1.22 m level platform which responded to the participants direction of motion and moved the tread belt opposite to this direction to keep the user centered to the treadmill platform (Figure 1b). Participants secured themselves to an overhead “positioning pole” via backpack straps which provided the operational boundaries and prevented participants from walking off the platform. Additionally, the omnidirectional treadmill allows users to bend over, kneel, and interact with virtual objects on the ground in VR. While on the omnidirectional treadmill, participants used the Vive Pro Eye VR headset and hand controllers (Vive, HTC Corp., Seattle, WA, USA) to complete the simulated EVA. The Vive Pro Eye was physically connected to a nearby computer (Alienware Aurora R16 [GPU = Nvidia RTX 4090], Dell Technologies, Round Rock, TX, USA).

Due to cybersickness and mechanical failure of the omnidirectional treadmill, testing was switched after the first *N* = 8 to a passive treadmill and sandbox configuration for the remaining *N* = 6 (Figure 1c). Participants walked on a self-powered, passive treadmill (Skillmill Connect, Technogym, Fairfield, NJ, USA) to simulate the traverses. The treadmill supports a range of resistances (1–10); however, participants were fixed to resistance settings ranging from 2 to 4, corresponding to the “low physical workload” condition in APACHE (Baughman et al., 2022). Additionally, a custom overhead harness (Tuff Tread, Conroe, TX, USA) was worn to mitigate injury from slips or falls while walking on the treadmill in VR (no gravitational offload was provided by the harness). Participants wore the Vive Ultimate Trackers (Vive, HTC Corp., Seattle, WA, USA) on their ankles to track their leg movement and enable translation in VR. Participants periodically dismounted the treadmill and entered the nearby sandbox—a 4.57 × 6.10 m space filled with ~7.60 cm deep of lunar regolith simulant—to complete the simulated geological sample identification task in VR. Due to the distance from the passive treadmill to the sandbox, a wired headset could not be used for this configuration. Rather, participants used the Meta Quest 3 VR headset (Quest, Meta Platforms Inc., Menlo Park, CA, USA) streaming wirelessly to a nearby computer. In both configurations, participants used their respective VR hand controllers to interact with the virtual environment and respond to the embedded cognitive tests.

### 2.3 VR EVA simulation

**Figure 2:**
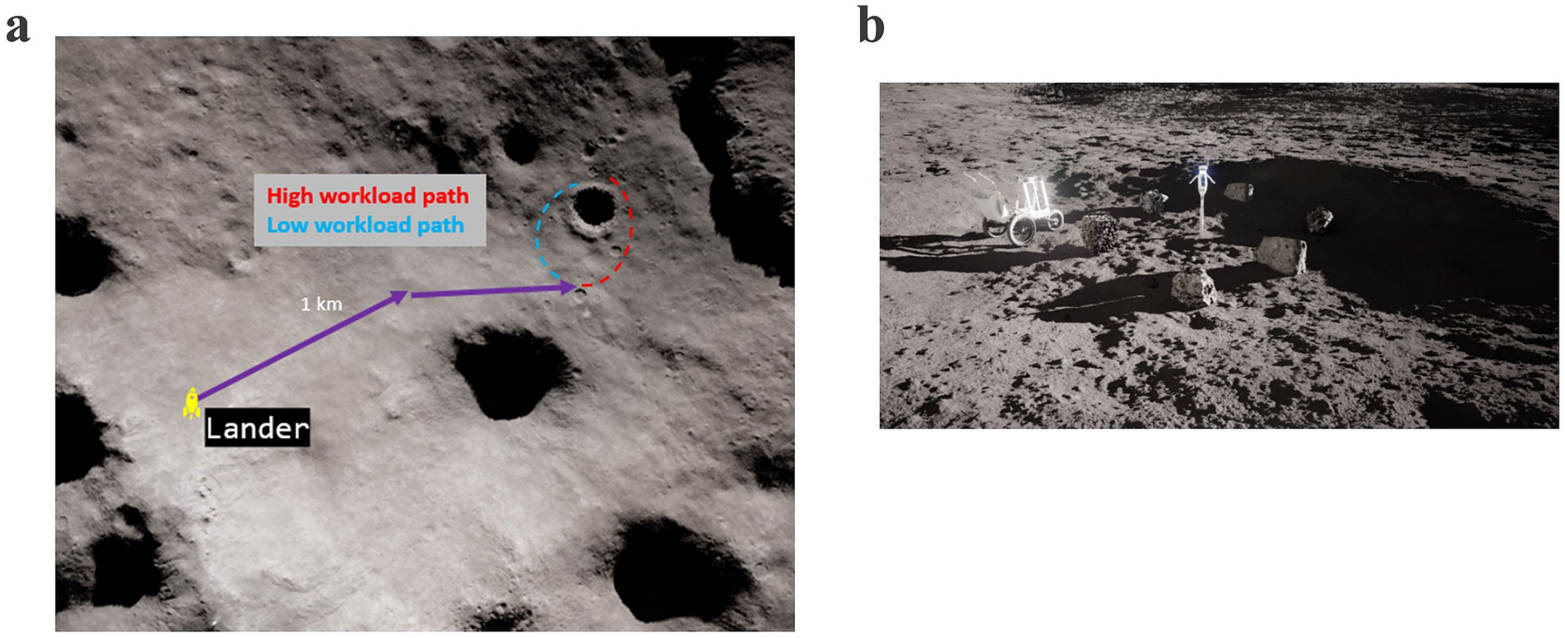
EVA simulation traverse path and example geology site. **(a)** Top-down view of traverse path to the large crater; **(b)** Proximal landscape view of a single example geology site in the APACHE cognitive workload study.

APACHE utilizes the Exploration Operation Support System (XOSS) for its VR simulations. XOSS is developed in collaboration with JSC’s Engineering Directorate and powered by Unreal Engine (UE5.4, Epic Games Inc., Cary, NC, USA). The virtual lunar environment contained ~4 × 4 km of explorable terrain near the Shackleton Crater on the Lunar South Pole. The virtual terrain was modeled using elevation data from the Lunar Reconnaissance Orbiter missions with a 5 m resolution accuracy. Smaller rocks, geology sites, and navigation poles were artificially placed on the surface to generate the EVA timelines. The simulated EVA started with the participant in their virtual spacesuit at the base of a lunar lander near their tools and tool cart. Participants traversed ~1 km from the lander to a large crater (Figure 2a) where four geology sites were located (Figure 2b). Depending on the test condition, participants were instructed to traverse clockwise or counterclockwise around the 120 m diameter crater, with each geology site equally spaced along the crater rim. To maintain a similar physical workload between test conditions, navigation poles were placed along the surface to guide participants to each geology site. On the omnidirectional treadmill, participants could change direction by simply walking in that direction; on the unidirectional passive treadmill, changing direction was accomplished by pressing a button on the hand controller. During the transition from the passive treadmill to the sandbox, participants were instructed to keep the VR headset on and switch to passthrough mode so that they could safely ingress the sandbox area. Once inside the sandbox, participants were quickly returned to the VR lunar landscape to maintain immersion in the simulation.

Each geology site was a collection of six lunar rocks, spaced relatively close together (4.57 × 6.10 m) so they would all fit within the limits of the APACHE sandbox. Participants were guided through the geological sample identification procedures by an intravehicular crewmember (IV) played by a member of the test team and completed the entire task in VR with their hand controllers. Participants were given a maximum of 30 min to complete geological sample identification and related tasks at each site. The overall timeline was restricted to 3 h, including time spent troubleshooting by the test team. Three EVA tasks, discussed next, were embedded in the overall EVA simulation.

### 2.4 Primary EVA task

Field geology was the primary task participants were asked to complete during EVA. Participants traversed to a series of four geology sites and within each site were summarily instructed to (1) visually search for and make geological callouts describing each rock’s size, shape, color, and texture; (2) use a handheld science instrument—the X-Ray Fluorescence (XRF) spectrometer—to determine the percentages of mineral compositions in each rock; (3) identify the top two rocks to sample from based on the instructed science objectives; (4) create chip samples from the rocks using a chisel and hammer; (5) take photos of each chip sample *in situ*; and (6) place each chip sample in a labeled sample bag.

**Figure 3:**
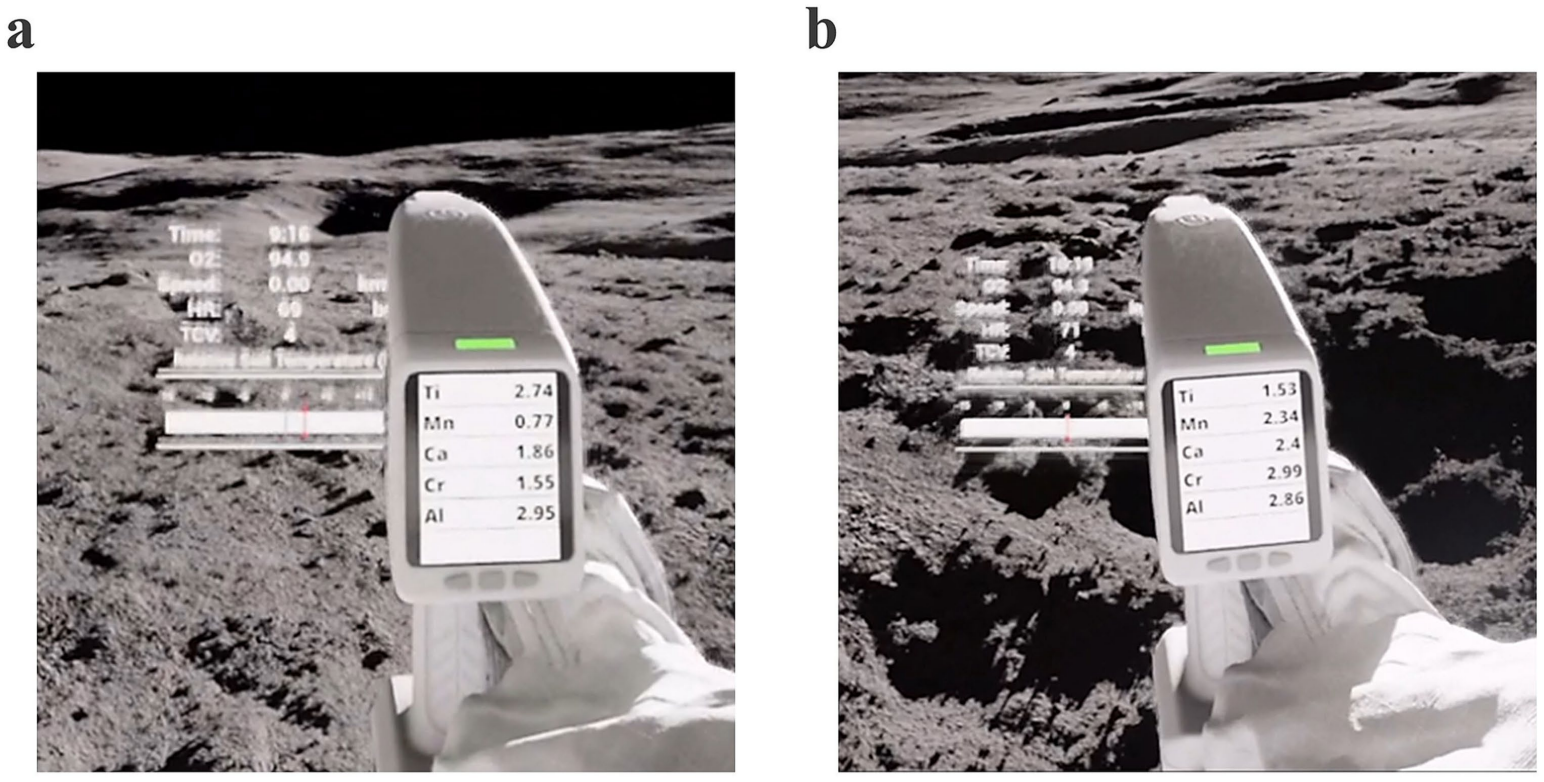
Primary EVA task of geology sampling of mineral rock composition using XRF. Images from participant training and familiarization. **(a)** The value of titanium in rock example A (Ti = 2.74%) is higher than **(b)** the value of titanium in rock example B (Ti = 1.53%); however, the overall weighted value of the minerals in rock example B is higher (B_ovr,A_ = 28.41 vs. B_ovr,B_ = 33.05). As a result, choosing rock example B would result in a higher quality geology sample.

Participants were briefed of the expected mineral compositions and their priority when identifying geological samples, from highest to lowest priority: (1) Titanium (Ti), (2) Manganese (Mn), (3) Calcium (Ca), (4) Chromium (Cr), (5) Aluminum (Al). The XRF spectrometer would always display these five minerals in the same order of decreasing priority. Although participants were instructed to seek out rock samples with high percentages in the most valuable mineral compositions, participants were asked to consider the *overall* priority of each rock sample based on all five of the mineral composition values available in the XRF scanner. As an example, taken from the participant training and familiarization, the value of Titanium in Rock Example A (Figure 3a, Ti = 2.74%) is higher than the value of Titanium in Rock Example B (Figure 3b, Ti = 1.53%); however, the *overall* weighted value of the minerals in Rock Example B is higher (B_ovr,A_ = (5 × 2.74) + (4 × 0.77) + (3 × 1.86) + (2 × 1.55) + (1 × 2.95) = 28.41 vs. B_ovr,B_ = (5 × 1.53) + (4 × 2.34) + (3 × 2.4) + (2 × 2.99) + (1 × 2.86) = 33.05). As a result, choosing Rock Example B would result in a higher quality geology sample. Participants were informed that they could only stow a total of eight samples across the four geology sites (two per site) during each EVA.

### 2.5 Secondary EVA task

**Figure 4:**
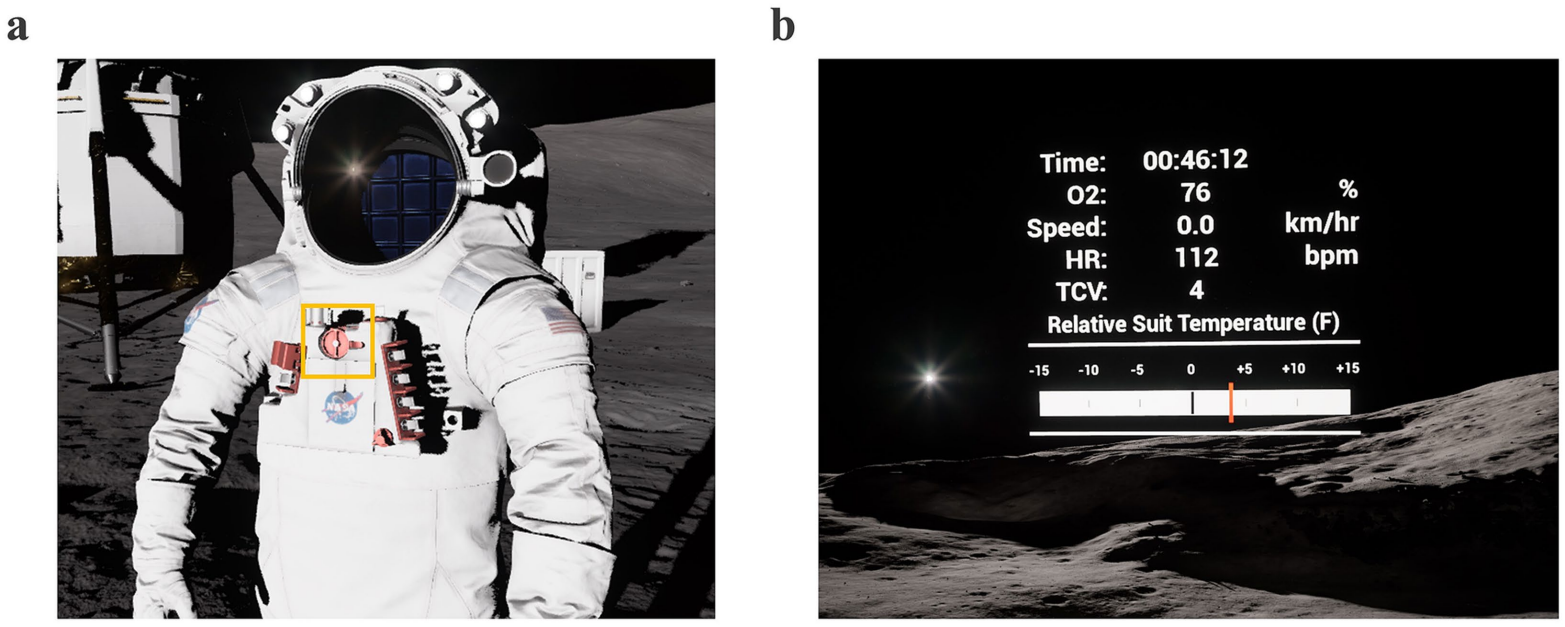
Secondary EVA task of monitoring suit temperature. **(a)** Spacesuit display and control unit (DCU) designated by orange box; **(b)** Relative suit temperature as shown in VR heads-up display.

Participants were also asked to continuously monitor and adjust their simulated spacesuit temperature throughout each EVA via a Thermal Control Valve (TCV) on the virtual spacesuit’s Display and Control Unit (DCU) (Ogilvie et al., 2023) (Figure 4a). Specifically, participants were instructed to keep their simulated spacesuit temperature—viewed as a red vertical line in the VR heads-up display (HUD)—as close to 0 as possible, with optimal performance defined as a relative suit temperature between −5 and +5 (Figure 4b). The simulated suit temperature was affected by the subject’s real-time heart rate (HR) from a Polar H10 strap and the current TCV suit setting (1–10) in VR. A higher heart rate was used to simulate a larger thermal load buildup due to metabolic processes, so the TCV needed to be set higher to remove more thermal heat. Vice versa, a high TCV setting combined with a decrease in HR resulted in a negative thermal load and a decrease in suit temperature. The maximum rate of change allowed (e.g., high HR and low TCV setting) for the simulated suit temperature was ±1 deg/min. Participants were not informed by the IV if their simulated suit temperature was outside the optimal bounds. Participants were instructed to self-monitor their simulated suit temperature throughout the EVA and adjust their virtual TCV setting accordingly to maintain optimal performance.

### 2.6 Tertiary EVA task

**Figure 5:**
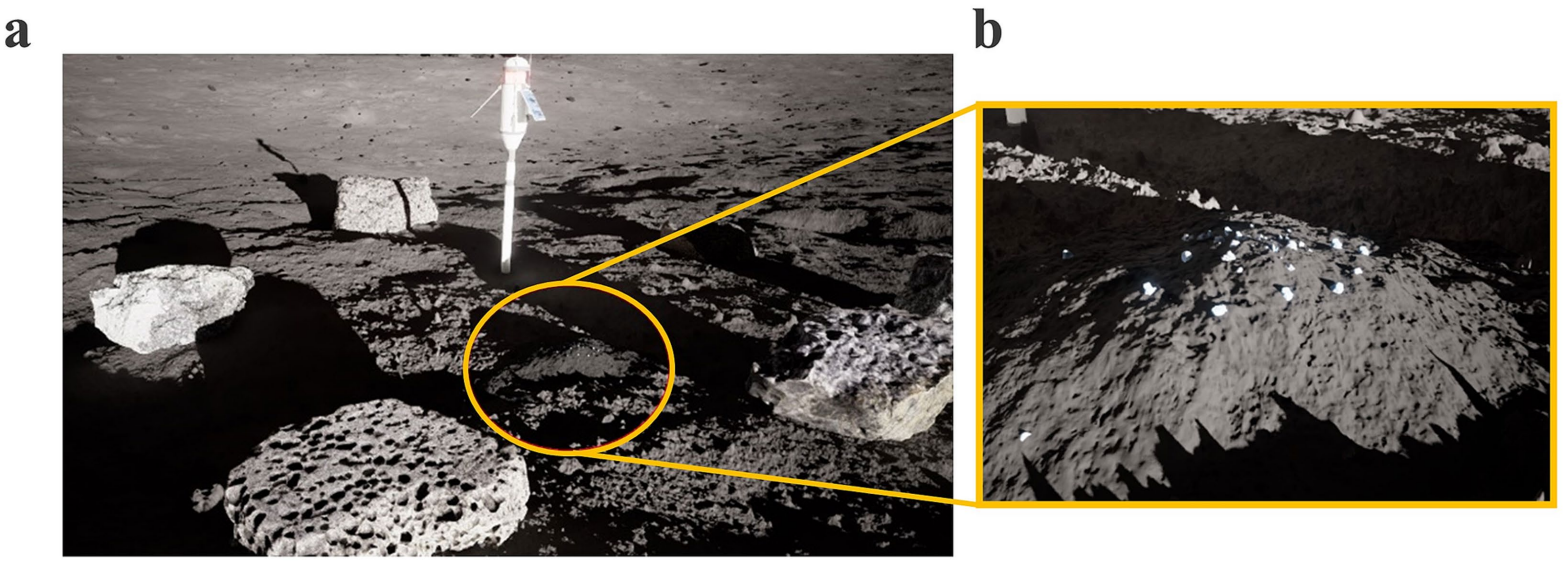
Tertiary EVA task of unexpected discovery of water ice crystals. Single example geology site with unexpected discovery designated by orange circle; magnified view of water ice crystals in inset.

In addition to the above tasks, participants were instructed at pre-briefing to “keep your eyes open for any unusual rocks or features beyond the ones targeted for geology sampling.” Unknown to participants, an “unexpected discovery” represented as water ice crystals buried in a small mound of lunar regolith (Figure 5) were placed within the bounds of two out of the four geology sites for each test condition.

### 2.7 Cognitive workload manipulation

Cognitive workload was manipulated during the primary EVA task of geological sample identification based on prior work suggesting geology to be one of the most cognitively demanding tasks astronauts execute during surface EVA (Anderson et al., 2026). During the EVA, participants were asked to conduct a compositional analysis on the identified rocks of interest using a VR-based handheld XRF spectrometer in determining each sample’s value based on a pre-defined rank order of types of minerals found in those samples. In the *Low Cognitive Workload* condition, the standard deviation of geological sample mineral composition values during XRF scanning was increased to make it *easier* for participants to identify samples highest in value. In the *High Cognitive Workload* condition, the standard deviation of mineral composition values during XRF scanning was decreased to make it *harder* to identify samples highest in value (Table 2). Cognitive workload conditions were blinded to participants and counterbalanced such that half of participants completed the High Cognitive Workload condition in the morning, while the other half of participants completed the High Cognitive Workload condition in the afternoon.

**Table 2:** Sample mineral composition values within geology sites across cognitive workload conditions.

Geology site	Low cognitive workload		High cognitive workload	
	Range	SD	Range	SD
1	17.2–38.4	±7.8	19.8–25.8	±2.2
2	17.0–37.1	±7.0	19.2–25.1	±2.2
3	16.9–34.7	±6.7	18.6–24.7	±2.3
4	16.5–33.1	±6.1	14.6–18.6	±1.5

Several steps were taken in the design of the simulated VR EVA to maintain equivalent physical workloads between the two experimental conditions. These included having participants in both conditions complete the same distance traverses and the same number of geology sites in both conditions, counterbalancing the order of high and low cognitive workload EVA simulations, and limiting the overall time that participants could spend at each geology site to approximately no more than 30 min.

### 2.8 Measures

**Figure 6:**
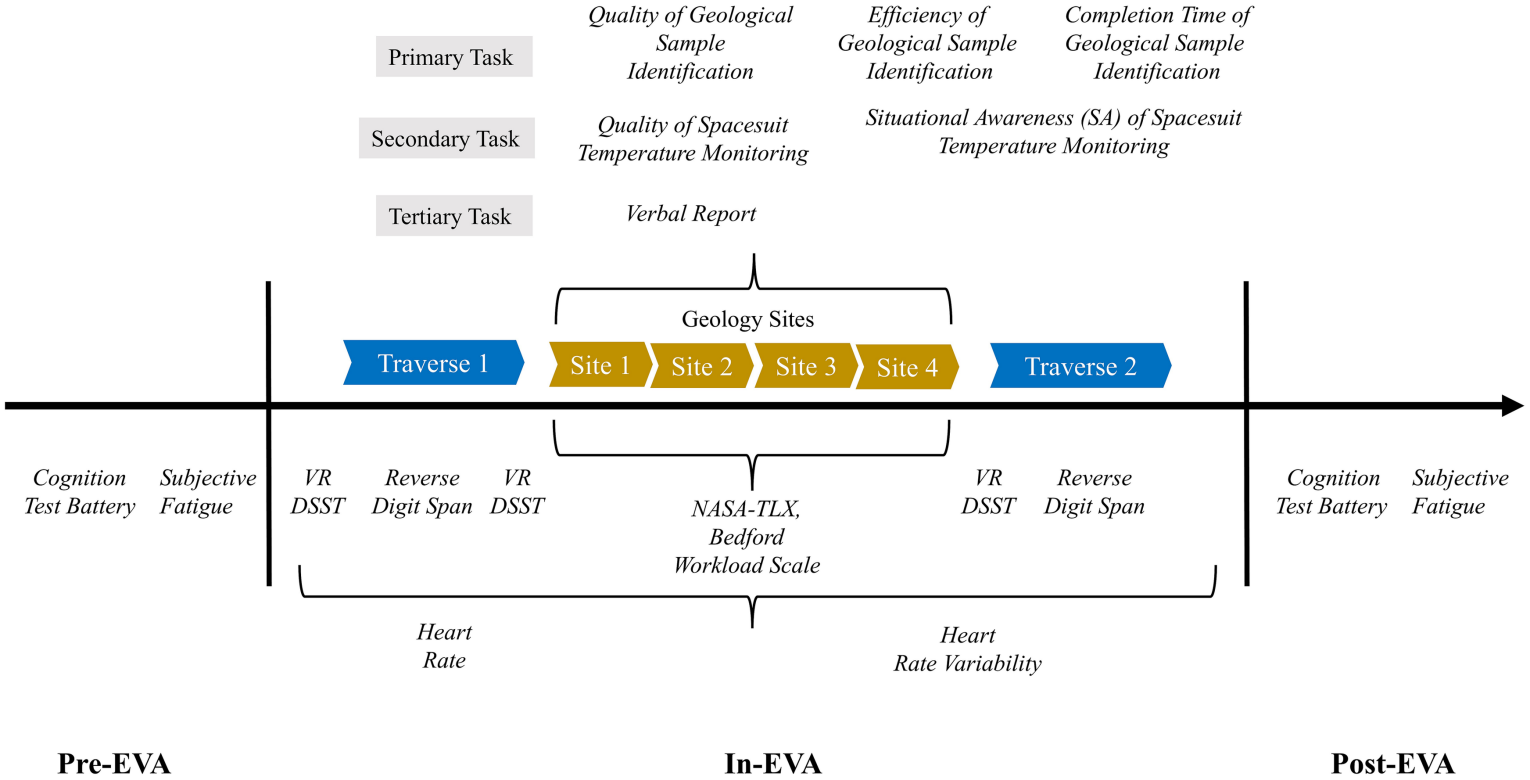
Test day measure administration timeline. VR DSST, virtual reality digit-symbol substitution test; NASA-TLX, NASA task load index.

Figure 6 shows the order measures were administered on test session days.

### 2.9 Cognitive workload

#### 2.9.1 NASA task load index (TLX)

The NASA-TLX (Hart and Staveland, 1988) survey is an assessment used to gain insights into six different dimensions of cognitive workload: mental demand, physical demand, temporal demand, performance, effort, and frustration. The NASA-TLX is a diagnostic and multi-dimensional tool that allows for the source of workloads to be localized, looking at the buildup of workload over time. In the present study, participants were asked to verbally report their TLX ratings for each dimension to the IV during the EVA after completing each geology site.

#### 2.9.2 Bedford workload scale

The Bedford Workload Scale (Casner and Gore, 2010), a modification of the Cooper-Harper rating scale, is a unidimensional rating scale designed to identify the operator’s spare mental capacity and cognitive workload level while completing a task. The level is assessed using a hierarchical decision tree that guides the operator through a 10-point rating scale, with each point accompanied by a description of the associated level of workload. The resulting metric is a single number rating on the scale between 1 and 10, where 1 corresponds to insignificant workload and 10 corresponds to the highest level of workload. Participants were asked to verbally report their Bedford ratings to the IV during the EVA after completing each geology site.

### 2.10 Cognitive performance

#### 2.10.1 VR digit-symbol substitution test (DSST)

The VR DSST is a VR adaptation of a paradigm used in the Wechsler Adult Intelligence Scale (WAIS-III) (Wechsler, 1997) to measure complex scanning and visual processing. The method of taking the VR DSST involved the participant pausing during their traverse to take the test. The participant viewed a popup overlay in VR of a key with various digits and symbols in their immediate field of view, with a pairing of one digit and one symbol appearing below the key. The participant was asked to respond by pressing the right controller if the number-symbol pairing is not in the key (i.e., the pairing is “false”) and the left controller if the pairing is in the key (i.e., the pairing is “true”). After each response, a new digit-symbol pairing appeared. The goal of the VR DSST is the same as the laptop-based DSST: to be as accurate and as fast as possible. Outcomes were average reaction time (RT), accuracy, and throughput (defined as % correct divided by average reaction time within a 90-s period). The VR DSST was completed (1) during the familiarization session (baseline), (2) after Traverse 1 prior to geology site 1, and (3) after geology site 4 prior to Traverse 2.

#### 2.10.2 Reverse digit span

A Reverse Digit Span task (Wechsler, 1997) assessing working memory was administered during EVA traverses to and from the lander ink VR. Participants were instructed to listen to a series of digits (1–9) presented in pseudorandomized order by the IV then to repeat the string of digits in backwards order. Successfully repeating the digits in the correct order resulted in the strings of digits becoming progressively longer (from a minimum of two digits to a maximum of nine digits), and thus more difficult, until the test was completed. The test was concluded after the participant got two strings of the same digit span length incorrect. The primary test metric was the digit span, or the longest sequence of numbers correctly reported by the participant. Participants completed the Reverse Digit Span while walking on the treadmill at two timepoints during each EVA: (1) at the start of the out traverse from the lander and (2) at the start of the in traverse back to the lander.

#### 2.10.3 Cognition test battery

The Cognition Test Battery (Basner et al., 2015) is a computerized test developed to assess a range of cognitive domains in high performing individuals, such as astronaut or astronaut-like populations. Cognition includes 10 subtests modeled after well-known neuropsychological tests assessing specific cognitive domains. The battery has 15 alternate versions that can be used to minimize practice effects with successive administrations. Speed and accuracy scores were calculated for each subtest in the Cognition Battery and published corrections to account for practice effects due to repeated administrations were applied (Basner et al., 2020). The Cognition Test Battery was administered at three timepoints during each Test Session: (1) prior to the start of the first EVA (Baseline), (2) immediately following the low workload condition EVA (Low Cognitive Workload), and (3) immediately following the high workload condition EVA (High Cognitive Workload).

### 2.11 Primary EVA task performance

#### 2.11.1 Quality of geological sample identification

The performance metric assessing the quality of geological sample identification during each EVA was the weighted sum of mineral composition values obtained through XRF scanning for each rock sample selected at geology sites. Specifically, the mineral composition values within each rock sample were multiplied by their corresponding weights, indicating value (Titanium = 5, Manganese = 4, Calcium = 3, Chromium = 2, Aluminum = 1). The resulting score was used to compare sample quality, with higher values indicating higher quality of geological sample identification.

#### 2.11.2 Efficiency of geological sample identification

At each geology site, subjects were allowed to scan the rocks with the XRF scanner as many times as they wanted prior to making a final decision for which rocks they wanted to sample. The frequency of XRF scans at each geology site was examined as a metric assessing the efficiency of geological sample identification. Specifically, the number of XRF scans from each geology site were counted and reverse scored, with higher values indicating more efficient geological sample identification via fewer XRF scans.

#### 2.11.3 Completion time of geological sample identification

The amount of time (in minutes) participants spent identifying geological samples at each site and during traverses was quantified to examine whether the cognitive workload manipulation affected completion time. Completion time at geology sites was limited to approximately 30 min.

### 2.12 Secondary EVA task performance

#### 2.12.1 Quality of spacesuit temperature monitoring

To assess performance of the secondary EVA task of self-spacesuit temperature monitoring, the absolute value of continuous relative suit temperatures across each EVA were aggregated by geology site and reverse scored, with higher values indicating higher quality of spacesuit temperature monitoring (via near-zero relative suit temperature).

#### 2.12.2 Situational awareness (SA) of spacesuit temperature monitoring

In addition to assessing the relative suit temperature maintained for the secondary EVA task, the frequency of TCV adjustments to operationalize SA of the secondary EVA task was also assessed. The number of TCV dial adjustments from each geology site was counted, with higher values indicating more SA of the suit temperature monitoring task.

### 2.13 Tertiary EVA task performance

#### 2.13.1 Verbal report

To assess performance on the tertiary EVA task, we documented whenever participants verbally reported the unexpected discovery (regardless of whether it was correctly identified as water ice) per geology site.

### 2.14 Physiological responding and fatigue

#### 2.14.1 Heart rate (HR) and heart rate variability (HRV)

HR data was collected continuously throughout each EVA using a Polar H10 HR strap worn across the chest. As part of safety monitoring for the study set by the NASA IRB, HR was monitored throughout each test session to ensure that HR did not exceed 85% of the participant’s age-predicted maximum HR, defined as 0.85 × (220 − age) (Fox, 1971; Nikolaidis et al., 2018). HR and heart rate variability (HRV, defined as the raw R-to-R Interval) data were processed using MATLAB R2024b (MathWorks, Natick, MA, USA) with custom-developed scripts for individual participant analyses. Data were time-synchronized to specific timeline steps in the transformed 24-h format. After synchronization, the data were aligned with task labels generated from the VR player’s actions based on in-sim events and the EVA timeline to enable task-based analysis. The event labels “Object Picked Up” (after “Station Arrive”) and “Object Dropped” (before “Station Leave”) were used to define the start and end of each geology task. Event labels were used solely for timing of tasks for the purposes of heart rate data analysis. Missing values and abrupt peaks in the data were removed and restored using interpolation. Abrupt peaks in the data were defined as observations more than three standard deviations away from the mean and were imputed using linear interpolation from adjacent data points in accordance with best practices (i.e., linear interpolation) and considering study-specific hardware and test limitations.

#### 2.14.2 Fatigue

Single item measures of subjective physical fatigue (“How physically tired are you feeling right now?” from 1 = Not at all to 7 = To a Very Great Extent) and mental fatigue (“How mentally tired are you feeling right now?” from 1 = Not at all to 7 = To a Very Great Extent) were collected pre- and post-EVA at each cognitive workload level in order to assess the effects of the cognitive workload manipulation on fatigue.

### 2.15 Statistical analysis

Robust linear mixed-effects model (LMM) regression analysis was used to examine the extent to which the cognitive workload condition influenced subjective and objective measures of cognitive workload and EVA performance. QQ plots of residuals generated for our models indicated a small degree of non-normality, particularly in the VR simulation data. Some extreme datapoints were created through technical issues experienced with the VR headset and treadmills. As a result, we chose robust LMM as it is more appropriate for extreme datapoints and violations of normality (Koller, 2016). Models were specified with cognitive workload condition (High, Low) predicting, in separate models, the following outcomes: self-reported cognitive workload (Manipulation Check), cognitive performance (Hypothesis 1), physiological responding and fatigue (Hypothesis 2), primary task EVA performance (Hypothesis 3), secondary task EVA performance (Hypothesis 4), or tertiary task EVA performance (Hypothesis 5). Model inputs included participant-specific intercepts, which were included as a random effect, and geology site and treadmill as fixed effects to examine temporal trends and to statistically control for any differences between the two treadmill configurations. We have reported any significant differences between treadmills observed from our models. For the robust LMM assessing the effect of cognitive workload on Reverse Digit Span performance, time of administration (Traverse 1, Traverse 2) was included to statistically control for any differences between the timing of administration of this measure For our analysis assessing changes in mental and physical fatigue due to the EVA, we specified a robust LMM with an interaction between time (Pre-EVA, Post-EVA) and cognitive workload condition (High, Low) predicting fatigue. Statistical significance was set to *α* = 0.05. All statistical analyses were performed using RStudio Version 4.4.1 (2024).

### 2.16 Data yield

Data yield is defined as the percentage data collection instances out of the total possible number of data collection instances in the protocol. Elective open text and branching logic to capture information only when applicable are not reflected in overall yield.

## 3 Results

### 3.1 Data yield

The data yield of our self-report and objective measures of cognitive workload and EVA performance was examined. Data yield was overall high across all measures (90.56%). Data yield was high for both primary (90.47%) and secondary (91.09%) measures. Measures with 100% data yield included the Reverse Digit Span, Cognition Test Battery, HR, pre- and post-EVA questionnaires, and self-reported cognitive workload assessments (Bedford Workload Scale, NASA-TLX), suggesting that they are operationally feasible measures in this context.

### 3.2 Cognitive workload manipulation check

**Figure 7:**
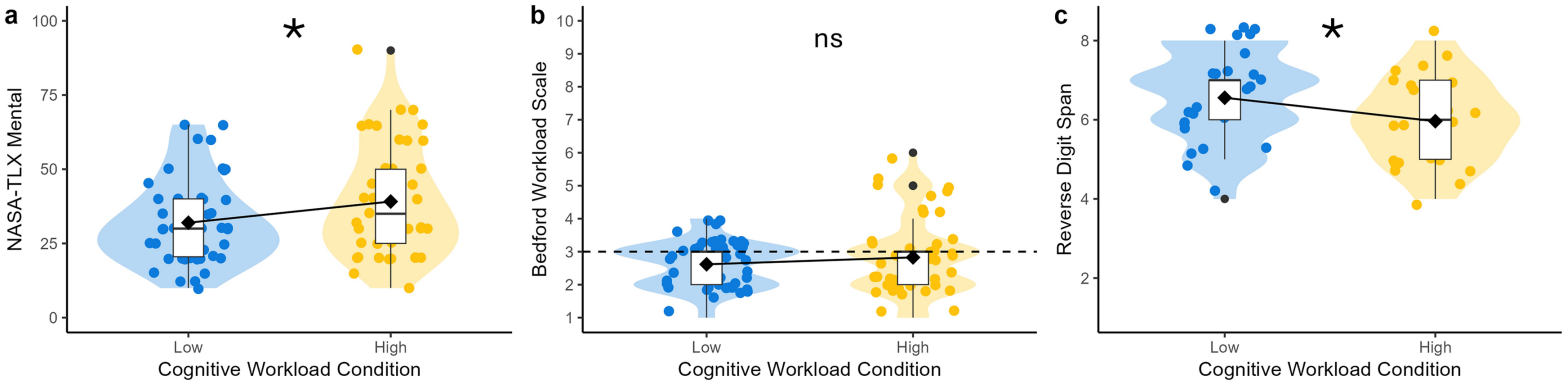
Cognitive workload and cognitive performance measures by cognitive workload condition. *p < 0.05. **(a)** NASA TLX mental demand dimension results by participant and geology site, with higher values indicating higher mental demand of the task; **(b)** Bedford workload scale results by participant and geology site, with higher values indicating less spare capacity; measurements for nominal tasks (critical or frequent) are limited to a Bedford workload scale rating of 3 or less per NASA-STD-3001, indicated as a dotted horizontal line; **(c)** Reverse digit span results by participant and time of administration (Traverse 1, Traverse 2), with higher values indicating better performance.

An examination of whether the cognitive workload manipulation was associated with self-reported cognitive workload—as measured by the NASA-TLX and Bedford Workload Scale—was conducted after each geology site as a manipulation check. Participants reported significantly higher TLX Mental scores following the high workload condition compared to the low workload condition geology sites: *β* = 4.62, SE = 2.14, 95% CI [0.36, 8.89], *t* = 2.16, *p* = 0.03 (Figure 7a). In contrast, TLX Physical, Temporal, Performance, Effort, and Frustration scores did not significantly differ by cognitive workload condition. Bedford Workload Scale scores also did not significantly differ by cognitive workload condition (Figure 7b; Table 3). This suggests that the cognitive workload manipulation was effective and increased subjective mental demand as measured by the NASA-TLX, although not the Bedford Workload Scale.

**Table 3:** Self-reported cognitive workload by cognitive workload condition.

Cognitive workload measure	Low cognitive workload *M* (SD)	High cognitive workload *M* (SD)	Results of robust linear mixed models				
			Estimate	Standard error	Confidence interval	Statistic	*p*
TLX mental	31.98 (13.96)	39.11 (18.09)	4.62	2.14	0.36–8.89	2.16	0.03
TLX physical	22.91 (9.69)	26.09 (12.88)	1.77	1.27	−0.77 to 4.30	1.39	0.17
TLX frustration	22.94 (19.28)	25.26 (22.85)	0.36	1.86	−3.33 to 4.05	0.19	0.85
TLX effort	33.21 (13.70)	34.39 (16.51)	−0.18	1.68	−3.52 to 3.15	−0.11	0.91
TLX performance	27.43 (17.41)	27.26 (16.95)	−1.24	1.38	−3.98 to 1.50	−0.90	0.37
TLX temporal	16.32 (10.26)	17.39 (15.23)	0.52	1.31	−2.07 to 3.12	0.40	0.69
Bedford workload	2.62 (0.71)	2.83 (1.18)	−0.10	0.10	−0.30 to 0.10	−0.98	0.33

In terms of differences in subjective workload ratings between treadmill configurations, participants on the passive treadmill reported significantly lower TLX Effort [*β* = −16.03, SE = 7.83, 95% CI = −31.60 to −0.47, *t* = −2.05, *p* = 0.04] and marginally lower TLX Frustration [*β* = −22.07, SE = 11.0, 95% CI = −43.93 to −0.20, *t* = −2.01, *p* = 0.05] scores than participants on the omnidirectional treadmill. Participants on the passive treadmill reported significantly lower Bedford Workload Scale scores than participants on the omnidirectional treadmill, *β* = −1.16, SE = 0.44, 95% CI = −2.03 to −0.29, *t* = −2.64, *p* = 0.01.

### 3.3 Cognitive performance

**Figure 8:**
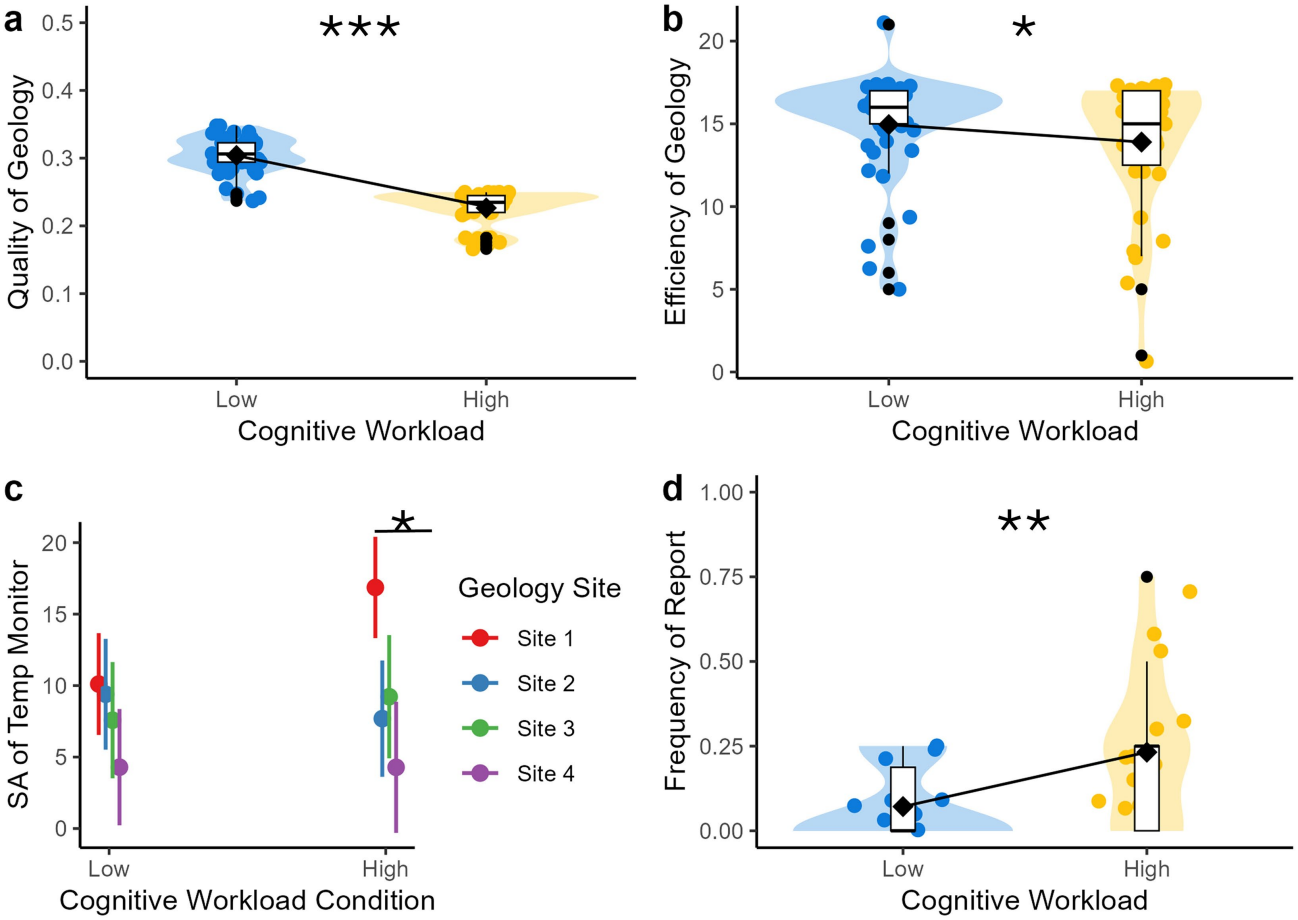
EVA performance metrics by cognitive workload condition. *p < 0.05, **p < 0.01, ***p < 0.001. **(a)** Quality of geology sampling as measured by the weighted sum of mineral composition values obtained through XRF scanning for each rock sample selected at geology sites. Higher values indicate higher quality of geology sampling; **(b)** Efficiency of geology sampling as measured by the frequency of XRF scans at each geology site, which were counted and reverse scored. Higher values indicate higher efficiency of geology sampling; **(c)** Interaction between cognitive workload and time (geology site) predicting situational awareness (SA) of suit temperature monitoring. Higher values indicate higher SA of suit temperature monitoring as measured by temperature control valve (TCV) adjustments. SA decreased more steeply from geology site 1–2 in the high compared to low workload condition, suggesting a steeper decline in SA to the secondary EVA task over time under conditions of high workload; **(d)** Number of times participants verbally reported the unexpected discovery (regardless of whether it was correctly identified as water ice) per geology site. Higher values indicate higher frequency of reporting the unexpected discovery.

Next, an examination of whether the cognitive workload manipulation was associated with measures of cognitive performance was conducted. Participants performed significantly worse on the Reverse Digit Span during the high workload compared to low workload condition, *β* = −0.50, SE = 0.21, 95% CI = −0.93 to −0.08, *t* = −2.37, *p* = 0.02 (Figure 8c). Time of administration (Traverse 1, Traverse 2) was not associated with Reverse Digit Span performance. This suggests that working memory as measured by the Reverse Digit Span was related to the cognitive workload demands of the EVA.

On the VR DSST, participants demonstrated significantly faster mean RT in both high workload [*β* = −74.05, SE = 20.17, 95% CI = −114.66 to −33.45, *t* = −3.67, *p* < 0.001] and low workload [*β* = −76.70, SE = 20.22, 95% CI = −117.41 to −35.99, *t* = −3.79, *p* < 0.001] conditions compared to baseline, but mean RT did not significantly differ between cognitive workload conditions. Similarly, VR DSST throughput improved in both high workload [*β* = 4.11, SE = 1.89, 95% CI = 0.31–7.91, *t* = 2.17, *p* = 0.03] and low workload [*β* = 6.36, SE = 1.89, 95% CI = 2.55–10.18, *t* = 3.36, *p* = 0.002] conditions compared to baseline, but did not significantly differ between cognitive workload conditions. This suggests that performance on an embedded task of complex scanning and visual tracking was improved by conducting the EVA but was not impacted by the cognitive workload demands of the EVA.

On the Cognition Test Battery, participants demonstrated marginally slower mean RT on the DSST after high workload condition compared to baseline (*β* = 49.10, SE = 24.05, 95% CI = 0.33–97.87, *t* = 2.04, *p* = 0.05), although mean RT did not differ between low and high cognitive workload conditions. Similarly, participants demonstrated significantly slower mean RT on the Psychomotor Vigilance Test (PVT) after high workload condition compared to baseline (*β* = 14.99, SE = 4.76, 95% CI = 5.34–24.65, *t* = 3.15, *p* = 0.003), although RT did not differ between low and high cognitive workload conditions. Proportion correct on the Emotion Recognition Test (ERT) was significantly lower after the low workload condition compared to baseline (*β* = −0.10, SE = 0.04, 95% CI = −0.18 to −0.01, *t* = −2.38, *p* = 0.02), although performance did not differ between low and high cognitive workload conditions. Finally, participants demonstrated significantly slower mean RT on the Fractal 2-Back (F2B) after high workload condition compared to baseline (*β* = 44.11, SE = 17.05, 95% CI [9.54, 78.69], *t* = 2.59, *p* = 0.01), although mean RT did not differ between low and high cognitive workload conditions. This suggests that performance on post-EVA tasks of complex scanning and visual tracking (DSST), vigilant attention (PVT), emotion recognition (ERT), and working memory (F2B) was negatively impacted by conducting the EVA but was not related to the cognitive workload demands of the EVA itself.

In terms of differences between treadmill configurations, participants on the passive treadmill performed significantly worse on the Reverse Digit Span compared to participants on the omnidirectional treadmill, *β* = −1.03, SE = 0.43, 95% CI = −1.90 to −0.16, *t* = −2.38, *p* = 0.02. Participants on the passive treadmill also had significantly slower mean RT on the Cognition Test Battery DSST compared to participants on the omnidirectional treadmill, *β* = 289.44, SE = 71.98, 95% CI = 143.45–435.43, *t* = 4.02, *p* < 0.001. Proportion correct on the Abstract Matching (AM) test was also significantly lower among participants on the passive compared to omnidirectional treadmill, *β* = −0.18, SE = 0.05, 95% CI = −0.27 to −0.08, *t* = −3.65, p < 0.001.

### 3.4 Primary EVA task performance

We next examined whether the cognitive workload manipulation was associated with participants’ performance on the primary EVA task of geology sampling. Participants performed significantly worse in terms of the quality of their geology sampling (total rock score) during the high workload condition compared to low workload condition: *β* = −0.08, SE = 0.006, 95% CI = −0.09 to −0.07, *t* = −14.82, *p* < 0.001 (Figure 8a). Participants also performed significantly worse in terms of the efficiency of their geology sampling (frequency of XRF scans) during the high compared to low workload condition, *β* = −0.89, SE = 0.41, 95% CI = −1.71 to −0.06, *t* = −2.14, *p* = 0.04 (Figure 8b).

The cognitive workload demands of the EVA did not influence how long participants spent at each geology site (Low Cognitive Workload Mean: 15.65 min, High Cognitive Workload Mean: 15.48 min). Four participants had individual geology site completion times that exceeded 30 min (range: 30.50–38.70 min). In each case, VR headset technical issues (e.g., system crash, arms not calibrating correctly) arising during either the first or second geology site led to completion times that exceeded the allotted time per site. Overall, these results suggests that high cognitive workload is related to the quality and efficiency of geology sampling, but not completion time of geology sampling, which may have been impacted by the schedule pressure of approximately 30 min per geology site.

Regarding any differences between treadmills, participants on the passive treadmill had significantly faster completion time of geology sites compared to participants on the omnidirectional treadmill, *β* = −3.11, SE = 1.19, 95% CI = −5.48 to −0.75, *t* = −2.61, *p* = 0.01.

### 3.5 Secondary EVA task performance

In terms of the secondary EVA task of suit temperature monitoring, the quality of participants’ suit monitoring (relative suit temperature) did not significantly differ between cognitive workload conditions. Participants’ SA of suit temperature monitoring (frequency of TCV adjustments) also did not differ between cognitive workload conditions. However, there was a significant decrease in SA of suit temperature monitoring over time, Site 2: *β* = −4.42, SE = 1.90, 95% CI = −8.21 to −0.63, *t* = −2.32, *p* = 0.023; Site 3: *β* = −4.67, SE = 1.99, 95% CI = −8.65 to −0.70, *t* = −2.34, *p* = 0.022; Site 4: *β* = −8.59, SE = 2.03, 95% CI = −12.65 to −4.54, *t* = −4.23, *p* < 0.001.

Given this result, a post-hoc interaction model (cognitive workload condition × time) was specified to examine whether the decrease in SA over time differed depending on the cognitive workload condition. In linear contrasts testing the difference between estimated marginal means, we found that there was significantly lower SA in geology Site 1 in the low (Estimated Marginal Mean = 10.04) compared to high (Estimated Marginal Mean = 16.80) cognitive workload condition, *β* = −6.76, SE = 2.34, *p* = 0.004. This suggests that although SA of suit temperature monitoring was initially higher in the high workload condition, there was a steeper decline in SA to the secondary task over time under conditions of high workload.

Testing differences between treadmills, participants on the omnidirectional treadmill had significantly higher quality of suit temperature monitoring compared to participants on the passive treadmill, *β* = −1.76, SE = 0.41, 95% CI = −2.62 to −0.91, *t* = −4.26, *p* < 0.001.

### 3.6 Tertiary EVA task performance

For the unexpected discovery task, contrary to our prediction, participants were more likely to report the water ice crystals in the high compared to low cognitive workload condition, *β* = 0.000007, SE = 0.000002, 95% CI = 0.00003 to 0.00001, *t* = 3.26, *p* = 0.002 (Figure 8d).

### 3.7 Physiological responding and fatigue

Mean HR while at geology sites was significantly higher in the high compared to low cognitive workload condition: *β* = 2.22, SE = 1.01, 95% CI = 0.20, 4.23, *t* = 2.19, *p* = 0.03. Average HRV was marginally lower while at geology sites in the high (*M* = 662.91, SD = 120.34) compared to low (*M* = 667.0, SD = 122.57) cognitive workload condition: *β* = 16.58, SE = 8.37, 95% CI = −0.37 to 33.53, *t* = 1.98, *p* = 0.05. HR significantly declined over the course of geology sites, Site 4: *β* = −4.07, SE = 1.52, 95% CI = −7.09 to −1.05, *t* = −2.69, *p* = 0.01. HRV significantly increased over the course of geology sites, Site 4: *β* = 42.62, SE = 12.91, 95% CI [16.48, 68.75], *t* = 3.30, *p* = 0.002. The change in HR and HRV due to cognitive workload suggests that physiological correlates of cognitive workload were associated with increased cognitive demand of the geological sample identification task. In contrast, the change in HR and HRV that we observed over time may suggest a reduction in stress over the course of the EVA due to increasing task familiarity.

Finally, subjective physical fatigue significantly increased from pre- to post-EVA [*β* = 0.86, SE = 0.35, 95% CI [0.15, 1.56], *t* = 2.45, *p* = 0.02], but was not influenced by cognitive workload condition. Similarly, subjective mental fatigue significantly increased from pre- to post-EVA [*β* = 1.06, SE = 0.52, 95% CI [0.31, 1.81], *t* = 2.85, *p* = 0.006], but was not influence by cognitive workload condition. Participants on the passive treadmill reported marginally lower physical fatigue than participants on the omnidirectional treadmill, *β* = −1.09, SE = 0.54, 95% CI = −2.17 to −0.003, *t* = −2.02, *p* = 0.05. Treadmill configuration did not influence subjectively reported mental fatigue.

## 4 Discussion

The purpose of the present study was to examine the extent to which cognitive workload in a simulated surface EVA task is related to subjective cognitive workload assessments, physiological correlates, and EVA performance outcomes. To test this, we manipulated cognitive workload during simulated surface EVA in the extended reality exploration surface analog APACHE. We experimentally manipulated the cognitive difficulty of geological sample identification by increasing or decreasing the standard deviation of geological sample mineral composition values, thus making it less or more cognitively demanding for participants to identify which rocks were highest in value and should be sampled. Our results are consistent with previous literature showing that virtual reality (VR) simulations can be used to successfully characterize the multi-dimensional aspects of cognitive workload (Dell’Agnola et al., 2020; Luque et al., 2024). Furthermore, our study demonstrates that an operationally-relevant task completed entirely within VR can be experimentally manipulated to increase the multi-dimensional aspects of cognitive workload, consistent with prior VR studies manipulating cognitive load (Elkin et al., 2024; Sankaranarayanan et al., 2020).

First, confirming the effectiveness of our experimental manipulation, we demonstrated that manipulating the cognitive difficulty of a specific surface EVA task, geological sample identification, was associated with increases in subjective cognitive workload, consistent with our hypothesis and confirming the effectiveness and specificity of our manipulation. In robust LMMs, participants reported significantly higher mental demand (NASA-TLX Mental Demand) in the high compared to low cognitive workload condition. This finding is consistent with the large literature in spaceflight and other operational settings (e.g., surgery, aviation) attesting to the sensitivity of the NASA-TLX to increases in task cognitive demand (Hart, 2006; Hu et al., 2016). In contrast, we did not see significant changes in the Bedford Workload Scale due to cognitive task difficulty. A possible explanation for this finding is the overall lower variability in responses that we observed for the Bedford Workload Scale due to its hierarchical decision tree format. Additionally, our results are consistent with prior experimental studies that have found cognitive workload manipulation effects on the NASA-TLX—but not the Bedford Workload Scale (Kim and Ji, 2013; Zheng et al., 2019). The diverging results on self-reported workload may suggest that the Bedford Workload Scale is less sensitive to experimental cognitive workload manipulations than the NASA-TLX and supports the small but significant effect that we observed with NASA-TLX ratings. Future simulated EVA research is needed to understand the sensitivity of these scales to EVA task characteristics.

Next, addressing Hypothesis 1, participants performed significantly worse on an embedded cognitive task of working memory—the Reverse Digit Span—in the high compared to low cognitive workload condition, consistent with our hypothesis. This finding is consistent with prior evidence of increased cognitive load associated with performance on this measure (Lo et al., 2021; Ruiz-Rabelo et al., 2015) and evidence from operational military populations of decrements in performance due to increased environmental stressors (Hocking et al., 2001). The increased working memory load of retaining XRF scans and mentally calculating the overall value of the rock samples during geology in the high cognitive workload condition likely contributed to decrements in performance on this test. In contrast, although there were decrements in VR DSST and Cognition Test Battery performance from baseline on subtests assessing working memory, vigilant attention, and complex scanning and visual tracking, performance did not significantly differ between the cognitive workload conditions.

Addressing Hypothesis 2, heart rate (HR) during geology was significantly higher, and heart rate variability (HRV) marginally lower, in the high compared to low cognitive workload condition, consistent with our hypothesis. Our findings are consistent with evidence attesting to the sensitivity of HR measures to increases in cognitive workload (Delliaux et al., 2019; Hughes et al., 2019). Evidence from other operational environments, such as surgery, further support the pattern we observed (i.e., increased HR and lower HRV) and their association with task performance (Solhjoo et al., 2019). Efforts in the design of our study to maintain equivalent physical workload between experimental conditions, coupled with our finding that NASA-TLX ratings of physical demand did not significantly differ between experimental conditions, suggests that the HR change we observed was due to the cognitive workload manipulation and not differences in physical workload. In contrast, subjectively reported physical and mental fatigue collected pre- and post-EVA was not influenced by the cognitive workload condition. This may be due to the distance in the timing of the fatigue assessment (before and after each EVA) from the experimental manipulation itself (only during geological sample identification).

Addressing Hypothesis 3, we found that participants performed significantly worse in terms of the quality and efficiency of their primary task of geology sampling during the high compared to low cognitive workload condition, consistent with our hypothesis and further confirming the effectiveness of our experimental manipulation on EVA performance. In contrast, completion time at geology sites was not influenced by cognitive workload condition, possibly due to the maximum duration of 30 min that we defined for each geology site.

Addressing Hypothesis 4, there was no significant difference in the quality of the secondary task of self-spacesuit temperature monitoring between cognitive workload conditions, counter to our hypothesis. However, in an interaction analysis we found that SA was initially higher in the high cognitive workload condition, yet there was a steeper decline in SA over time under conditions of high workload. From a dual-task paradigm perspective, our finding that increased cognitive load of geological sample identification did not impact spacesuit temperature monitoring suggests that the effect of completing multiple tasks during EVA may negatively impact only the primary task at hand, although this effect is likely to depend on the interaction of the specific tasks conducted (Plummer and Eskes, 2015). Our result that SA declined over time during the EVA is also consistent with a time-on-task paradigm of cognitive fatigue (Mackworth, 1968; Mangin et al., 2022), with decrements in performance over time as cognitive fatigue increases throughout the EVA. This finding is further consistent with the results of a previously conducted surface EVA cognitive task analysis (Anderson et al., 2026), in which SA was highlighted by astronauts and EVA subject matter experts as a cognitive capability at risk of decrements during surface EVA.

Finally, addressing Hypothesis 5, participants were significantly more likely to verbally report the unexpected water ice crystals in the high compared to low cognitive workload condition, counter to our hypothesis. It is possible that the elevated cognitive workload in the high cognitive workload condition increased participants’ general SA to unexpected features of the environment during EVA. This would be consistent with our finding that SA toward the secondary EVA task of spacesuit temperature monitoring was initially elevated in the high workload condition, but declined more steeply over time. These findings suggest that, at least initially, the high cognitive workload condition may have increased attentional aspects of SA during our study. Prior findings in studies of automobile drivers indicating declines in SA as cognitive workload increases (Castro et al., 2019; Sahoo et al., 2025) suggests that further increases in cognitive workload during EVA may lead to similar decrements in SA over time as cognitive fatigue increases. Future research is needed to understand the thresholds at which cognitive workload leads to decrements in attention and SA during EVA.

There are several limitations of the present study to consider. First, participants did not experience physical gravity offloading while in the APACHE facility during the study. However, the APACHE lunar VR simulation applies lunar gravity to character models and objects, including the geology tools and rock samples that were a focus of our cognitive workload manipulation. Future research studies are needed to investigate how the experience of partial gravity influences cognition during surface EVA. Second, technical issues with the omnidirectional treadmill necessitated a switch to a passive treadmill and sandbox configuration partway through data collection. Although we statistically controlled for differences in treadmill type in our models, and reported where there were any statistically significant differences between treadmills, future research is needed to understand the effects of treadmill configuration on cognitive workload. Third, participants completed testing in a shirtsleeves environment with a virtual spacesuit but did not wear an actual spacesuit; future research is needed to understand how cognitive workload is impacted by spacesuits, as would be the case during EVA. Next, for experimental control, the present study manipulated cognitive workload in a single EVA task type (geological sample identification) but was not able to consider differing levels of cognitive workload across multiple EVA task types. In addition, a resting baseline of HR was not defined; therefore, all HR analyses represent HR data in each cognitive workload condition rather than a change from baseline. HRV data may be less accurate or harder to interpret in dynamic motion studies compared to resting or static conditions. This may help explain the only marginal difference in HRV that was observed between low and high cognitive workload conditions.

Taken together, our results demonstrate a relationship between the cognitive demands of a surface EVA task and subjective cognitive workload assessments, cognitive performance outcomes, physiological correlates, and EVA performance outcomes. Translating our findings to future surface EVA conducted on the lunar surface, our results suggest that the cognitive workload associated with geological sample identification may have important impacts on astronaut physiology and EVA task performance and should be an important consideration for EVA schedulers and monitoring for critical mission tasks. The added environmental challenges of real-world lunar surface EVA, including risks to astronaut safety, spacesuit ambulation, and partial gravity, are likely to further add to the cognitive workload increases we characterized in our simulated EVA study. Prior findings that Apollo astronauts during lunar EVA did not experience metabolic rates as high as predicted by ground-based simulations (Waligora et al., 1975) may not equally apply to the cognitive workload associated with lunar EVA.

The results from this study may inform specific NASA standards and guidelines for exploration missions (e.g., NASA Standard 3001). The cognitive workload data in the present study will be integrated into NASA’s Crew State and Risk Model (CSRM) along with other physiological models to predict crew risk states related to cognitive and physical fatigue during EVA (Hoffmann et al., 2025). Crew risk states indicated by CSRM will ultimately inform EVA biomedical decision support tools. Further utilizing innovative ground-based EVA simulation environments to investigate the physical and cognitive demands of surface EVA will better inform modeling efforts, standards and guidelines, and ultimately mission success in future visits to the Moon and Mars.

## Data Availability

The raw data supporting the conclusions of this article will be made available by the authors, without undue reservation.

## Footnotes

1
https://www.nasa.gov/directorates/esdmd/hhp/eva-operations-risk/

## References

[ref2] Anderson S. R. Jorge M. N. Bell S. T. (2026). Identifying cognitive capabilities required for optimal surface extravehicular activity performance. NPJ Microgravity. 12. doi: 10.1038/s41526-025-00545-1 PMC12775082 41353210

[ref3] Ari C. D’Agostino D. P. Bharwani S. Rehsi A. Moss S. Schmer-Galunder S. . (2020). Changes in individual and team cognition in high stress extreme underwater saturation environment under intense workload. FASEB J. 34, 1–1. doi: 10.1096/fasebj.2020.34.s1.09601

[ref4] Baddeley A. D. (2017). “Exploring the central executive” in Exploring working memory (Cambridge, UK: Routledge), 253–279.

[ref5] Basner M. Hermosillo E. Nasrini J. Saxena S. Dinges D. F. Moore T. M. . (2020). Cognition test battery: adjusting for practice and stimulus set effects for varying administration intervals in high performing individuals. J. Clin. Exp. Neuropsychol. 42, 516–529. doi: 10.1080/13803395.2020.1773765, 32539487 PMC7375457

[ref6] Basner M. Savitt A. Moore T. M. Port A. M. McGuire S. Ecker A. J. . (2015). Development and validation of the cognition test battery for spaceflight. Aerosp. Med. Hum. Perform. 86, 942–952. doi: 10.3357/amhp.4343.2015, 26564759 PMC4691281

[ref7] Baughman A. Kim K. J. Suri K. Abercromby A. (2022). “Assessments of physiology and cognition in hybrid-reality environments (APACHE)--physical workload approximation” in 51st international conference on environmental systems, Saint Paul, MN.

[ref8] Casner S. M. Gore B. F. (2010). Measuring and evaluating workload: a primer. NASA technical memorandum, 216395, National Aeronautics and Space Administration Ames Research Center, California, USA.

[ref9] Castaldo R. Melillo P. Bracale U. Caserta M. Triassi M. Pecchia L. (2015). Acute mental stress assessment via short term HRV analysis in healthy adults: a systematic review with meta-analysis. Biomed. Signal Process. Control 18, 370–377. doi: 10.1016/j.bspc.2015.02.012

[ref10] Castro S. C. Strayer D. L. Matzke D. Heathcote A. (2019). Cognitive workload measurement and modeling under divided attention. J. Exp. Psychol. Hum. Percept. Perform. 45, 826–839. doi: 10.1037/xhp0000638, 30998070

[ref11] Coan D. A. Miller M. J. (2023). Extravehicular activity (EVA) & human surface mobility (HSM) program (EHP) joint EVA & HSM test team (JETT) field test 3 (JETT3) report. Houston, Texas, USA: National Aeronautics and Space Administration Johnson Space Center.

[ref12] Dell’Agnola F. Momeni N. Arza A. Atienza D. (2020). Cognitive workload monitoring in virtual reality based rescue missions with drones. In: Chen, J.Y.C., and Fragomeni, G. (eds) Virtual, augmented and mixed reality. Design and interaction. HCII 2020. Lecture Notes in Computer Science, vol 12190. Springer, Cham. doi: 10.1007/978-3-030-49695-1_26

[ref13] Della Sala S. Baddeley A. Papagno C. Spinnler H. (1995). Dual-task paradigm: a means to examine the central executive. In J. Grafman, K. J. Holyoak, and F. Boller (eds.), Structure and functions of the human prefrontal cortex, New York Academy of Sciences, New York, USA. 161–171. 10.1111/j.1749-6632.1995.tb38137.x 8595023

[ref14] Delliaux S. Delaforge A. Deharo J.-C. Chaumet G. (2019). Mental workload alters heart rate variability, lowering non-linear dynamics. Front. Physiol. 10:565. doi: 10.3389/fphys.2019.00565, 31156454 PMC6528181

[ref15] Dunn J. Benson E. Norcross J. Newby N. (2022). Evidence report: risk of injury and compromised performance due to EVA operations. National Aeronautics and Space Administration Johnson Space Center, Houston, Texas, USA.

[ref16] Elkin R. L. Beaubien J. M. Damaghi N. Chang T. P. Kessler D. O. (2024). Dynamic cognitive load assessment in virtual reality. Simul. Gaming 55, 755–775. doi: 10.1177/10468781241248821

[ref17] Fox S. III (1971). Physical activity and the prevention of coronary heart disease. Ann. Clin. Res. 3, 404–432. 4945367

[ref18] Gathmann B. Schiebener J. Wolf O. T. Brand M. (2015). Monitoring supports performance in a dual-task paradigm involving a risky decision-making task and a working memory task. Front. Psychol. 6:142. doi: 10.3389/fpsyg.2015.00142, 25741308 PMC4330715

[ref19] Golding J. F. (2006). Predicting individual differences in motion sickness susceptibility by questionnaire. Pers. Individ. Differ. 41, 237–248. doi: 10.1016/j.paid.2006.01.012

[ref20] Green P. MacLeod C. J. (2016). SIMR: an R package for power analysis of generalized linear mixed models by simulation. Methods Ecol. Evol. 7, 493–498. doi: 10.1111/2041-210x.12504

[ref21] Hart S. G. (2006). “NASA-task load index (NASA-TLX); 20 years later” in Proceedings of the human factors and ergonomics society annual meeting. Sage CA: Los Angeles, CA: Sage publications.

[ref22] Hart S. G. Staveland L. E. (1988). “Development of NASA-TLX (task load index): results of empirical and theoretical research” in P. A. Hancock and N. Meshkati (eds.) Human Mental Workload, (Amsterdam: North Holland Press), 52: 139–183.

[ref23] Hertzum M. (2021). Reference values and subscale patterns for the task load index (TLX): a meta-analytic review. Ergonomics 64, 869–878. doi: 10.1080/00140139.2021.1876927, 33463402

[ref24] Hocking C. Silberstein R. B. Lau W. M. Stough C. Roberts W. (2001). Evaluation of cognitive performance in the heat by functional brain imaging and psychometric testing. Comp. Biochem. Physiol. A Mol. Integr. Physiol. 128, 719–734. doi: 10.1016/s1095-6433(01)00278-1, 11282316

[ref25] Hoffmann B. T. Kim K. J. Frisco D. Cooper L. Kirkley C. Garbino A. . (2025). “Development of the crew state and risk model for autonomous biomedical physiology state monitoring and prediction during exploration extravehicular activities” in 54th international conference on environmental systems (ICES). Prague, Czech Republic.

[ref26] Hu J. S. Lu J. Tan W. B. Lomanto D. (2016). Training improves laparoscopic tasks performance and decreases operator workload. Surg. Endosc. 30, 1742–1746. doi: 10.1007/s00464-015-4410-8, 26173550

[ref27] Hughes A. M. Hancock G. M. Marlow S. L. Stowers K. Salas E. (2019). Cardiac measures of cognitive workload: a meta-analysis. Hum. Factors 61, 393–414. doi: 10.1177/0018720819830553, 30822151

[ref28] Kim J. Y. Ji Y. G. (2013). A comparison of subjective mental workload measures in driving contexts. J. Ergon. Soc. Korea 32, 167–177. doi: 10.5143/jesk.2013.32.2.167

[ref29] Koller M. (2016). robustlmm: an R package for robust estimation of linear mixed-effects models. J. Stat. Softw. 75, 1–24. doi: 10.18637/jss.v075.i06 32655332 PMC7351245

[ref30] Kumle L. Võ M. L.-H. Draschkow D. (2021). Estimating power in (generalized) linear mixed models: an open introduction and tutorial in R. Behav. Res. Methods 53, 2528–2543. doi: 10.3758/s13428-021-01546-0, 33954914 PMC8613146

[ref31] Lee K.-M. Kang S.-Y. (2002). Arithmetic operation and working memory: differential suppression in dual tasks. Cognition 83, B63–B68. doi: 10.1016/s0010-0277(02)00010-0, 11934408

[ref32] Lo M. Lin Y.-X. Li Y.-J. (2021). Cognitive workload in an auditory digit span task when memory span is in the neighborhood of seven items. J. Psychophysiol. 36, 49–64. doi: 10.1027/0269-8803/a000282

[ref33] Logie R. H. (2011). The functional organization and capacity limits of working memory. Curr. Dir. Psychol. Sci. 20, 240–245. doi: 10.1177/0963721411415340

[ref34] Luque F. Armada V. Piovano L. Jurado-Barba R. Santamaría A. (2024). Understanding pedestrian cognition workload in traffic environments using virtual reality and electroencephalography. Electronics 13:1453. doi: 10.3390/electronics13081453

[ref35] Mackworth J. F. (1968). Vigilance, arousal, and habituation. Psychol. Rev. 75:308. doi: 10.1037/h0025896, 4875885

[ref36] Mangin T. Audiffren M. Lorcery A. Mirabelli F. Benraiss A. André N. (2022). A plausible link between the time-on-task effect and the sequential task effect. Front. Psychol. 13:998393. doi: 10.3389/fpsyg.2022.998393, 36389536 PMC9643466

[ref38] Nikolaidis P. T. Rosemann T. Knechtle B. (2018). Age-predicted maximal heart rate in recreational marathon runners: a cross-sectional study on Fox's and Tanaka's equations. Front. Physiol. 9:226. doi: 10.3389/fphys.2018.00226, 29599724 PMC5862813

[ref39] Ogilvie R. Miller S. Rundle T. (2023). “Space suit portable life support system thermal control valve ball design” in 52nd international conference on environmental systems (ICES). Calgary, Canada.

[ref40] Plummer P. Eskes G. (2015). Measuring treatment effects on dual-task performance: a framework for research and clinical practice. Front. Hum. Neurosci. 9:225. doi: 10.3389/fnhum.2015.00225, 25972801 PMC4412054

[ref41] Rai B. Kaur J. Foing B. H. (2012). Stress, workload and physiology demand during extravehicular activity: a pilot study. N. Am. J. Med. Sci. 4, 266–269. doi: 10.4103/1947-2714.97205, 22754877 PMC3385362

[ref42] Richter C. Braunstein B. Winnard A. Nasser M. Weber T. (2017). Human biomechanical and cardiopulmonary responses to partial gravity–a systematic review. Front. Physiol. 8:583. doi: 10.3389/fphys.2017.00583, 28860998 PMC5559498

[ref43] Ruiz-Rabelo J. F. Navarro-Rodriguez E. Di-Stasi L. L. Diaz-Jimenez N. Cabrera-Bermon J. Diaz-Iglesias C. . (2015). Validation of the NASA-TLX score in ongoing assessment of mental workload during a laparoscopic learning curve in bariatric surgery. Obes. Surg. 25, 2451–2456. doi: 10.1007/s11695-015-1922-1, 26459432

[ref44] Sahoo P. Bain A. J. Biondi F. N. (2025). Investigating the interplay between cognitive workload and situation awareness during full driving automation. Theor. Issues Ergon. Sci. 26, 457–477. doi: 10.1080/1463922X.2024.2446851

[ref45] Sankaranarayanan G. Odlozil C. A. Wells K. O. Leeds S. G. Chauhan S. Fleshman J. W. . (2020). Training with cognitive load improves performance under similar conditions in a real surgical task. Am. J. Surg. 220, 620–629. doi: 10.1016/j.amjsurg.2020.02.002, 32107012 PMC8054609

[ref46] Schlotman T. E. Cox L. I. McGrath T. M. Baughman A. J. Estep P. N. Siders B. A. . (2023). “A preliminary assessment of physical demand during simulated lunar surface extravehicular activities” in 2023 IEEE aerospace conference. Big Sky, Montana, USA.

[ref47] Schubert C. Lambertz M. Nelesen R. Bardwell W. Choi J.-B. Dimsdale J. (2009). Effects of stress on heart rate complexity—a comparison between short-term and chronic stress. Biol. Psychol. 80, 325–332. doi: 10.1016/j.biopsycho.2008.11.005, 19100813 PMC2653595

[ref48] Shahini F. Zahabi M. (2022). Effects of levels of automation and non-driving related tasks on driver performance and workload: a review of literature and meta-analysis. Appl. Ergon. 104:103824. doi: 10.1016/j.apergo.2022.103824, 35724471

[ref49] Solhjoo S. Haigney M. C. McBee E. van Merrienboer J. J. Schuwirth L. Artino A. R. Jr. . (2019). Heart rate and heart rate variability correlate with clinical reasoning performance and self-reported measures of cognitive load. Sci. Rep. 9:14668. doi: 10.1038/s41598-019-50280-3, 31604964 PMC6789096

[ref50] Waligora J. M. Hawkins W. Humbert G. Nelson L. Vogel S. Kuznetz L. (1975). Apollo experience report: assessment of metabolic expenditures. National Aeronautics and Space Administration Johnson Space Center, Houston, Texas, USA.

[ref51] Wechsler D. (1997). WAIS-III administration and scoring manual: Psychological Corporation. San Antonio, Texas, USA.

[ref52] Zheng Y. Lu Y. Jie Y. Fu S. (2019). Predicting workload experienced in a flight test by measuring workload in a flight simulator. Aerospace Med. Human Perf. 90, 618–623. doi: 10.3357/AMHP.5350.2019, 31227035

